# Impact of storage-induced chemical changes on heterocyclic amine formation in chicken breast meat

**DOI:** 10.1016/j.fochx.2026.104136

**Published:** 2026-06-24

**Authors:** Jeong-Uk Eom, Nayeem Mia, Jin-Kyu Seo, Han-Sul Yang

**Affiliations:** a Institute of Agriculture and Life Science, Gyeongsang National University, 501 Jinju-daero, Jinju-si, Gyeongsangnam-do 52828, Republic of Korea; b Division of Applied Life Science (BK21Four), Gyeongsang National University, 501 Jinju-daero, Jinju-si, Gyeongsangnam-do 52828, Republic of Korea

**Keywords:** Chicken breast meat, Heterocyclic amines, Storage conditions, Protein carbonylation, Volatile compounds

## Abstract

Although thermal processing generates carcinogenic heterocyclic amines (HCAs) in cooked meat, previous research has largely focused on cooking-stage variables, leaving the biochemical impact of the pre-cooking storage phase poorly understood. During storage, lipid and protein oxidation products accumulate along with proteolytic shifts, which can alter the precursor matrix required for the Maillard reaction. This study evaluates how varying storage conditions influence subsequent HCA formation in chicken breast. Refrigerated storage for 14 days promoted oxidation and proteolysis, substantially altering volatile carbonyl and free amino acid profiles. These changes established a reactive precursor environment in which accumulated carbonyls and amino acids coexisted, potentially contributing to the formation of most individual HCAs during subsequent cooking. In contrast, frozen storage effectively suppressed oxidative degradation and reduced overall HCA accumulation, except for PhIP. Ultimately, monitoring storage-phase chemical indicators enables HCA prediction and mitigation, offering a proactive approach to food safety management.

## Highlights

• Storage temperatures and durations drive biochemical shifts in chicken breast.
• Refrigeration accumulates oxidative products and alters free amino acid profiles.
• Storage-formed chemical matrices favor HCA synthesis during cooking.
• Frozen storage suppresses oxidative pathways but mitigates HCA species selectively.
• Storage-induced biochemical shifts predict subsequent HCA formation risks.

## 1 Introduction

Storage conditions are a critical factor in determining the biochemical state of meat prior to thermal processing. During storage, lipid oxidation and proteolysis occur as concomitant processes; lipid oxidation leads to the accumulation of reactive electrophiles, such as malondialdehyde (MDA) and 4-hydroxynonenal (4-HNE), while proteolysis induces protein carbonylation and significantly modifies the profile of free amino acids (FAAs) (Domínguez et al., 2019; Zhang et al., 2013). The rate and pattern of these oxidative and degradative reactions vary significantly with storage temperature and duration. While refrigerated storage is characterized by sustained enzymatic and oxidative activity, frozen storage introduces distinct shifts, including cellular structural damage due to ice crystal formation and accelerated oxidation during the thawing phase (Leygonie et al., 2012; Lund et al., 2011). Consequently, these divergent storage modalities – refrigeration versus freezing – establish unique chemical landscapes in meat before it ever encounters heat.

The chemical environment established during storage is closely correlated with the formation of heterocyclic amines (HCAs) during subsequent cooking. It is well established that the concentration of key HCA precursors – including FAAs, creatine, and lipid oxidation products – undergoes continuous shifts during storage (Domínguez et al., 2019). These precursor profiles, in turn, significantly influence the amount of HCAs generated (Adeyeye, 2020; Geng et al., 2024). Given that major HCA species, such as PhIP, IQ, and MeIQx, are synthesized through specific Maillard-based pathways involving nitrogenous precursors and carbonyl compounds, variations in the storage-induced chemical matrix may differentially modulate the formation patterns of each HCA type (Puangsombat et al., 2012). From this perspective, the specific carbonyl species accumulated, and the altered FAA profiles serve as preconditioning factors associated with HCA outcomes, even when identical cooking parameters are applied.

Despite its significance, systematic investigation into the role of storage-induced chemical shifts as a contributing factor in HCA formation remains sparse. Current HCA research has predominantly concentrated on cooking-stage variables, such as temperature, duration, and cooking methods (Rahman et al., 2014). Meanwhile, storage has largely been examined through the lens of quality degradation or oxidation inhibition, with few studies evaluating it as a modulating condition for HCA synthesis (Adeyeye & Ashaolu, 2021; Wang et al., 2021). Consequently, research integrating storage temperature and duration as independent variables to analyze the nexus between precursor evolution and HCA yields is extremely limited.

To address these knowledge gaps, the present study posits that the chemical environment established during the storage stage is closely associated with the potential for subsequent HCA reactions during cooking, rather than viewing HCA formation as an isolated result of thermal processing. To test this hypothesis, we comprehensively analyzed the volatile compound and FAA compositions of chicken breast stored under varying temperatures and durations, and evaluated their quantitative relationships with HCA yields. Ultimately, this research aims to demonstrate that biochemical transitions during storage are associated with the potential for subsequent HCA formation, which may further contribute to the generation of pro-carcinogenic compounds.

## 2 Materials and methods

### 2.1 Sample preparation and cooking procedure

The experiments were conducted in 9 independent batches (*n* = 9) on different dates under identical environmental conditions to ensure statistical reproducibility. For each batch, 2 kg of whole chicken breasts from broilers slaughtered on the same day were purchased from a commercial butcher shop located in Jinju, South Korea. Each batch represented a distinct biological pool to ensure treatment independence. Upon transport to the laboratory, all visible fat and connective tissues were manually trimmed. The trimmed chicken breasts were then randomly allocated to four treatment groups of 500 g each, including refrigerated storage at 4 °C for 1 and 14 days, and frozen storage at −20 °C for 1 and 14 days. All samples were vacuum-packaged and stored at their respective temperatures.

Before HCA analysis, all frozen samples were completely thawed at 4 °C for 24 h to synchronize the initial sample temperature with the refrigerated group (4 °C). For standardized cooking, 200 g of each minced sample was formed into a uniform shape using a Petri dish to tightly control the thickness and surface area. Before roasting, the griddle was pre-calibrated by monitoring the surface temperature at 200 °C, 250 °C, and 300 °C using a laser infrared thermometer (Bluebird, BO-350, China). For thermal processing, the synchronized samples were cooked on the preheated electric griddle at 200 °C for a total of 12 min (6 min on each side), and the core temperature of each patty was determined using a probe thermometer (RS components, Beauvais, France) to record the endpoint of cooking at 72 °C. This standardized cooking procedure and extensive 24-h thawing regime were strictly controlled across all 9 independent replicates to minimize potential variations in thermal histories and moisture dynamics among the treatment groups.

### 2.2 pH

After mixing 3 g of a minced muscle sample with 27 mL of distilled water, it was homogenized for 30 s using a Polytron homogenizer (T25 basic; IKA Labortechnik, Selangor, Malaysia). All homogenized samples were analyzed with a pH meter (MP230; Mettler Toledo, Greifensee, Switzerland). Four pH measurements were recorded for each sample, and the average value was used. The pH meter was calibrated at 20 °C using three standard buffers of pH 4.0, 7.0, and 9.0.

### 2.3 Lipid and protein oxidation determination

Lipid oxidation was determined using a modified method based on Salih et al. (1987). Briefly, 3 g of sample was homogenized with 27 mL of 3.86% perchloric acid using a homogenizer (T25, IKA, Staufen, Germany) at 14,000 rpm for 30 s. The homogenate was then allowed to stand in a temperature-controlled dark chamber for 1 h to facilitate lipid extraction. The extracted mixture was filtered through Whatman No. 1 filter paper, and 2 mL of the filtrate was mixed with 2 mL of 20 mM thiobarbituric acid (TBA). The reaction mixture was incubated at room temperature for 15 h, after which absorbance was measured at 531 nm using a spectrophotometer (Cary 60, Agilent Technologies, Santa Clara, USA). The TBA value was calculated by multiplying the absorbance by a conversion factor of 5.58 and expressed as mg malondialdehyde (MDA)/kg meat.

The carbonyl content was determined by modifying the method described by Jeong et al. (2023). Briefly, 3 g of chicken breast was homogenized with cold 0.15 M potassium chloride (KCl) using a homogenizer (ULTRA-TURRAX T-25, IKA, Staufen, Germany) at 10,000 rpm for 30 s. Subsequently, 1 mL of cold 10% trichloroacetic acid (TCA) was added to the homogenate, followed by centrifugation at 5000*g* for 5 min (LaboGene 1736R, LABOGENE, Lillerød, Denmark). After discarding the supernatant, 0.4 mL of 5% sodium dodecyl sulfate (SDS) was added to the pellet, and the mixture was subjected to high-speed sonication (VC750, Sonic & Materials Inc., Newtown, CT, USA) to ensure complete dissolution. The sample was divided into two aliquots. To one aliquot, 0.8 mL of 2 M hydrochloric acid (HCl) containing 10 mM dinitrophenylhydrazine (DNPH) was added, while 0.8 mL of 2 M HCl without DNPH was added to the other aliquot as a control. The samples were incubated at room temperature for 30 min. Thereafter, 0.2 mL of cold 20% TCA was added, and the mixture was centrifuged at 5000*g* for 5 min. The supernatant was removed, and the pellet was washed with ethanol-ethyl acetate (1,1, *v*/v) to remove excess DNPH. This washing step was repeated until residual DNPH was completely eliminated. After washing, 1.5 mL of 6 M guanidine-HCl prepared in 20 mM phosphate buffer (pH 6.5) was added to both DNPH-treated and control samples, and the mixtures were incubated at room temperature for 24 h. Following incubation, the samples were centrifuged at 10,000*g* for 10 min, and absorbance was measured at 280 nm and 370 nm using a microplate spectrophotometer (BioTek, Winooski, VT, USA) with 96-well UV plates (SPL, Pocheon-si, Korea). The protein carbonyl content (nmol/mg protein) was quantified using the molar extinction coefficient of 22,000 M^−1^ cm^−1^ and a microplate protein correction factor of 0.43 with a unit conversion factor of 10^6^. The carbonyl content was calculated as follows:

$$\frac{A_{370}-A_{370\left(\mathrm{blank}\right)}}{22,000\times \left[A_{280}-\left(A_{370}-A_{370\left(\mathrm{blank}\right)}\right)\right]\times 0.43}\times 10^{6}$$

### 2.4 Free amino acids

Free amino acids were extracted according to the method of Seo et al. (2024) with minor modifications. Briefly, 3 g of sample and 27 mL of deionized water were homogenized for 30 s and 10 mL of 10% TCA solution was added and shaken at 250 rpm for 1 h at room temperature. After an hour, centrifugation was performed at 10,000*g* for 10 min and the supernatant was filtered using Whatman No.1. Finally, it was filtered with a 0.2 μm syringe filter, transferred to a 1.5 mL vial, and used for analysis. A 20 μL aliquot was injected into an amino acid analyzer (Biochrom 30, Biochrom, Cambridge, UK) equipped with a high-performance sodium ion-exchange column. Separated amino acids were automatically derivatized via post-column reaction with ninhydrin within the analyzer system, and detected spectrophotometrically at 570 nm and 440 nm. Quantification was performed using the standard with 40 amino acids (Physiology standard, Biochrom) via the manufacturer's integration software. The results were expressed as mg of free amino acid/100 g of sample.

### 2.5 Determination of creatine

Creatine content was determined based on the methods of Polak et al. (2009) with minor modifications. Samples were rapidly frozen in liquid nitrogen and stored at −80 °C until analysis. Approximately 0.1 g of finely ground chicken breast was homogenized for 5 min using a homogenizer (T25, IKA, Staufen, Germany) in 20 mL of 30 g/L trichloroacetic acid (TCA) to precipitate proteins and extract creatine. The homogenate was filtered through Whatman No. 1 filter paper to remove insoluble material. An aliquot of 20 mL of the filtrate was defatted with 10 mL of diethyl ether and allowed to stand for 10 min to achieve phase separation. After removal of the upper ether layer, 4 mL of the defatted extract was transferred to a reaction tube. Subsequently, 2 mL of diacetyl solution (0.2 g/L) and 2 mL of 1-naphthol solution (25 g/L) prepared in 20 g/L sodium hydroxide were added. The reaction mixture was incubated at 40 °C for 5 min, and absorbance was measured at 520 nm using a spectrophotometer (Cary 60, Agilent Technologies, Santa Clara, CA, USA). Creatine content was quantified based on the absorbance at 520 nm.

### 2.6 Heterocyclic amines analysis

After the cooking, the samples were lyophilized using a freeze-dryer (Alpha 1–2 LDplus; Martin Christ, Osterode am Harz, Germany) to prepare them for HCA analysis. HCAs extraction was performed according to Parvin et al. (2023) with minor modifications. Freeze-dried chicken breast samples (30 g) were mixed with 120 mL of 2 M sodium hydroxide and pre-incubated at 37 °C for 30 min. The suspension was homogenized using a high-speed sonication system (VC750, S/N: 98166AR-10-17; Sonics & Materials Inc., Newtown, CT, USA) for 15 min to obtain a uniform slurry and then blended with 300 g of Celite 545. HCAs were extracted using an Extrelut NT column (Extrelut NT packaging material; Merck, Darmstadt, Germany) with 600 mL of dichloromethane at a flow rate of 2 mL/min. The extract was filtered through Whatman No. 1 filter paper and sequentially purified using a preconditioned PRS cartridge (strong cation-exchange sorbent) followed by a C18 cartridge (0.5 g sorbent). The PRS cartridge was preconditioned with 6 mL of 0.1 N hydrochloric acid, and HCAs were eluted with 15 mL of 0.1 M HCl/methanol at a flow rate of 3 mL/min, followed by washing with 2 mL of distilled water. The eluate was transferred to the preconditioned C18 cartridge, washed with 5 mL of ultrapure water, and dried. HCAs were then eluted with 1.2 mL of methanol containing 10% ammonia into 1.8 mL vials. The eluates were evaporated under nitrogen at 50 °C for 30 min using a multi-chamber gas flow concentrator (Barkey GmbH & Co. KG, Leopoldshöhe, Germany). The dried residues were reconstituted in 100 μL of methanol containing TriMeIQx as an internal standard, and 10 μL was injected for analysis. HCAs were analyzed using HPLC (Dionex Ultimate 3000, Thermo Fisher Scientific, Sunnyvale, CA, USA) equipped with a diode-array UV detector and an autosampler. Chromatographic separation was achieved on a Shim-pack VP-ODS column (150 × 4.6 mm, 5 μm; Shimadzu, Kyoto, Japan). The mobile phases consisted of 50 mM ammonium acetate (pH 3.6) (A) and acetonitrile (B). The gradient program was as follows: 10% B initially, increased to 60% at 15 min, 95% at 17 min, held until 20 min, returned to 10% at 21 min, and maintained until 28 min at a flow rate of 1 mL/min. The column temperature was maintained at 35 °C. Identification of HCAs was performed by comparing retention times and UV spectra with reference standards, and quantification was conducted using TriMeIQx as an internal standard (10 ng/g). To ensure a consistent and stable analytical baseline independent of varying moisture content during storage, the final HCA concentrations were quantified and expressed strictly on a dry-weight basis (ng/g dry matter).

### 2.7 Measurement of volatile compounds

#### 2.7.1 Headspace solid-phase microextraction

Approximately 2.5 g of chicken breast was mixed with 5 mL of 25% (*w*/*v*) sodium chloride solution to promote the release of volatile compounds. The mixture was homogenized for 1 min using a high-speed homogenizer and transferred to a 20 mL glass vial. An internal standard (2-methyl-3-heptanone, 0.05 μg/mL in hexane, 1 μL) was added, and the vial was immediately sealed to prevent volatile loss. Headspace solid-phase microextraction (HS-SPME) was carried out using a DVB/CAR/PDMS fiber (50/30 μm; Sigma-Aldrich, Germany). Prior to extraction, samples were equilibrated at 60 °C for 15 min. The SPME fiber was then exposed to the headspace for 30 min at the same temperature under continuous agitation. After extraction, the fiber was inserted into the GC injection port and thermally desorbed for 7 min. The fiber was conditioned at 270 °C for 30 min before initial use and reconditioned at 270 °C for 15 min between analyses.

#### 2.7.2 GC–MS operating conditions

Volatile compounds were analyzed using a GC–MS system composed of an Agilent 7890B gas chromatograph coupled with an Agilent 5973C mass selective detector and equipped with a PAL autosampler (Agilent Technologies, Santa Clara, CA, USA). Helium (99.999% purity) served as the carrier gas at a constant flow rate of 1.0 mL/min. Injection was performed in pulsed splitless mode using a deactivated SPME liner (0.75 mm i.d., Agilent), with the injector temperature set at 250 °C. Separation was achieved on an HP-INNOWAX capillary column (30 m × 0.25 mm × 0.25 μm; Agilent). The oven temperature program was as follows: 40 °C for 3 min, increased to 120 °C at 4 °C/min, then to 220 °C at 8 °C/min, and finally to 250 °C at 20 °C/min with a 5 min hold. The transfer line temperature was maintained at 280 °C. Mass spectrometry was operated in electron ionization (EI) mode at 70 eV, with ion source and quadrupole temperatures set at 230 °C and 150 °C, respectively. Mass spectra were acquired over an *m*/*z* range of 50–450.

### 2.8 Method validation

The analytical performance parameters of the method are summarized in Table 1, focusing on linearity, sensitivity, precision, accuracy, and matrix interference. Linearity was evaluated using a seven-point calibration curve (0.5–100.0 ng/g). The limits of detection (LOD) and quantification (LOQ) were determined based on a signal-to-noise (S/N) ratio of 3 and 10, respectively. Accuracy and matrix effects (ME) were evaluated using fortified meat matrices. Recovery was calculated by comparing the peak areas of target HCAs from pre-extraction fortified samples with those spiked post-extraction (*n* = 9). Matrix effects were assessed by comparing the peak areas of post-extraction spiked extracts against pure solvent standards. Intra-day repeatability (*n* = 3) and inter-day reproducibility (*n* = 9, evaluated over three consecutive days) were expressed as relative standard deviations (RSD, %). Peak identification was confirmed by cross-referencing retention times and DAD-UV spectra with certified reference standards (≥ 98% purity).

**Table 1:** Analytical method validation parameters for the quantification of 10 heterocyclic amines using HPLC.

Compound	Calibration (ng/g)	Regression Equation	Linearity (*R*^*2*^)	LOD (ng/g)	LOQ (ng/g)	Recovery (%)	Repeatability (Intra-day, RSD %)	Reproducibility (Inter-day, RSD %)	Matrix Effect (%)
IQ	0.5–100.0	y = 6314.15x + 344.20	0.9997	0.04	0.12	72.4	3.2	4.1	−4.2
IQX	0.5–100.0	y = 10,728.69xx + 511.45	0.9999	0.05	0.15	68.9	4.1	5.3	−6.8
MeIQx	0.5–100.0	y = 6351.79x + 298.10	0.9998	0.03	0.09	78.1	2.8	3.8	−2.1
7,8-DiMeIQx	0.5–100.0	y = 4795.54x + 210.85	0.9998	0.06	0.18	64.2	4.9	6.1	−7.5
4,8-DiMeIQx	0.5–100.0	y = 5100.06x + 245.30	0.9998	0.05	0.15	66.5	4.3	5.9	−5.9
Harman	0.5–100.0	y = 9305.18x + 412.50	0.9999	0.02	0.06	81.3	1.9	2.9	−1.1
PhIP	0.5–100.0	y = 3027.85x + 138.40	0.9998	0.03	0.09	75.6	3.4	4.5	−3.4
Norharman	0.5–100.0	y = 8406.19x + 395.20	0.9999	0.02	0.06	79.8	2.1	3.2	−1.3
AαC	0.5–100.0	y = 2493.61x + 165.70	0.9999	0.08	0.24	58.4	5.2	7.2	−9.1
MeAαC	0.5–100.0	y = 2015.44x + 112.90	0.9999	0.11	0.33	56.1	5.8	7.8	−9.5

Recovery, mean values obtained from spiked meat matrices (*n* = 9).

Repeatability and Reproducibility, expressed as relative standard deviation (RSD, %) measured intra-day (*n* = 3) and inter-day over three consecutive days (*n* = 9), respectively.

Matrix Effect, calculated by comparing peak areas of standards spiked post-extraction with pure solvent standards.

### 2.9 Statistical analysis

The experiment was conducted using a completely randomized block design with a factorial arrangement of treatments (2 storage temperatures × 2 storage periods). A total of 9 independent batches prepared on different days were used as independent biological replicates, with each batch serving as the experimental unit. All chemical and instrumental analyses for each batch were performed in triplicate. Storage temperature, storage period, and their interaction were treated as fixed effects, whereas batch was included as a random effect in the model using a variance components covariance structure. Prior to analysis, data normality and model assumptions were verified using the Shapiro-Wilk test, along with residual diagnostics, to ensure model adequacy. Statistical analyses were performed using the GLIMMIX procedure of SAS software (version 9.4, SAS Institute Inc., Cary, NC, USA). Least squares means were estimated, and when significant effects were detected, differences among treatment means were compared using the Tukey-Kramer adjustment at *P* < 0.05. Multivariate analyses, including principal component analysis (PCA), variable importance in projection (VIP) scores, and cross-validation, were performed using MetaboAnalyst 6.0. The robustness and predictability of the PLS-DA model were evaluated using cross-validation (cumulative *R*^*2*^ and *Q*^*2*^ values), and the model was further validated against overfitting using a 100-time permutation test (*P* < 0.05).

## 3 Results and discussion

### 3.1 Changes in pH and creatine during refrigerated and frozen storage

**Fig. 1:**
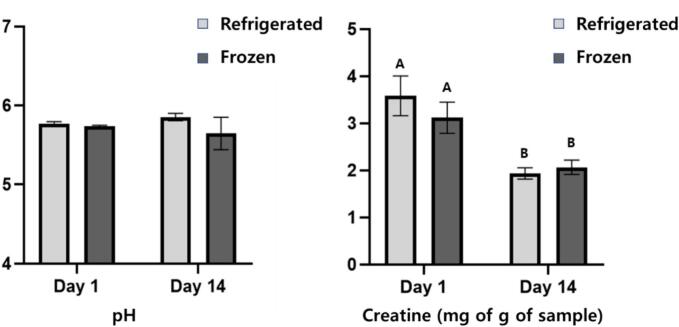
Changes in pH and creatine of chicken breast during refrigerated and frozen storage. Values are expressed as mean ± SEM. Different letters indicate significant differences among storage condition × storage period (*P* < 0.05).

The changes in pH and creatine content were analyzed to evaluate the fundamental chemical environment of chicken breast and indicators of HCA precursors under different storage conditions. As shown in Fig. 1, the pH remained within the range of 5.7–5.9 across storage durations (1 and 14 days) and temperatures (refrigeration and freezing), with no significant differences observed. This indicates that pH changes were limited under the current experimental conditions, suggesting that subsequent chemical variations were unlikely to be directly induced by pH fluctuations. In contrast, creatine content decreased significantly as storage duration increased. Compared to Day 1, the creatine content at Day 14 decreased at both storage temperatures, although no significant difference was observed between the temperatures.

Generally, creatine is known to convert into creatinine during thermal processing, thereby contributing to the formation of HCAs (Adeyeye et al., 2025). The results of this study demonstrated that creatine was depleted during refrigerated or frozen storage, which is consistent with previous findings (Jung et al., 2025). These results imply that storage duration has a greater effect on creatine content than the specific storage method. This phenomenon may be attributed to the freeze-concentration effect caused by drip loss during thawing, which creates an environment that facilitates the conversion of creatine to creatinine as temperature increases (Leygonie et al., 2012). Consequently, while both pH and creatine are recognized as factors affecting HCA generation during cooking, our findings suggest that pH is unlikely to be a defining factor in the pre-existing HCA formation environment. Instead, the observed changes in creatine levels suggest its potentially crucial role in subsequent HCA formation.

### 3.2 Lipid and protein oxidation during storage

**Fig. 2:**
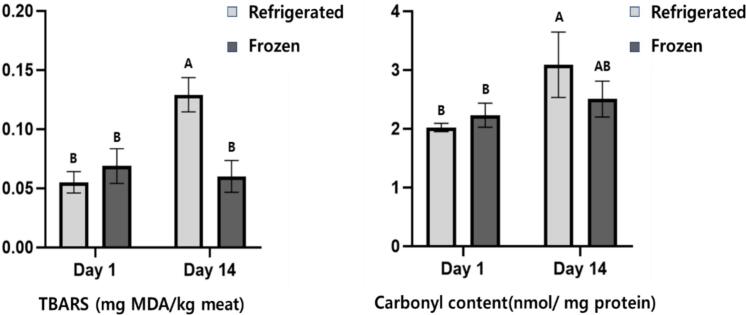
Changes in TBARS and carbonyl content of chicken breast during refrigerated and frozen storage. Values are expressed as mean ± SEM. Different letters indicate significant differences among storage condition × storage period (*P* < 0.05).

Previous studies on HCA formation have primarily focused on the reactions between free amino acids and creatine; however, the indirect role of carbonyl compounds generated during oxidative processes has recently been proposed (Deng et al., 2024; Zamora & Hidalgo, 2020). Accordingly, this study simultaneously evaluated lipid and protein oxidation to elucidate changes in HCA formation during storage. The results are presented in Fig. 2, where significant interaction effects were observed for both lipid oxidation (TBARS) and protein oxidation (carbonyl content) (*P* < 0.05). TBARS values significantly increased in the refrigerated storage group by Day 14, reaching levels more than twice those on Day 1. In contrast, changes in TBARS values under frozen storage conditions were relatively limited, indicating that freezing effectively inhibited lipid oxidation, although this oxidative suppression did not translate into a universal reduction of all HCA types. Carbonyl content, an indicator of protein oxidation, also increased significantly during storage. Notably, the carbonyl content on Day 14 under refrigeration was significantly higher than on Day 1 (*P* < 0.05). These findings demonstrate that storage temperature is a major factor in regulating lipid oxidation rate.

These results are generally consistent with trends reported in previous literature. According to prior studies, lipid oxidation progresses rapidly with increasing storage time at refrigeration temperatures, whereas frozen storage effectively suppresses it (Domínguez et al., 2019). On the other hand, protein oxidation tends to increase gradually over time regardless of the storage temperature, and carbonyl compounds have been proposed as cumulative indicators of protein oxidation during long-term storage (Estévez, 2011; Lund et al., 2011). The oxidative products accumulated during storage are expected to serve as substrates that accelerate the formation of specific HCAs during thermal processing. It has been reported that samples with active lipid oxidation show a more pronounced generation of HCAs, such as PhIP (Zamora & Hidalgo, 2020). This is because reactive carbonyl compounds produced during lipid and protein oxidation facilitate the formation of Strecker aldehydes, which are key intermediates in the Maillard reaction. Aldehydes derived from lipid and protein oxidation directly provide the carbonyl groups necessary for forming the ring structures that constitute the core skeleton of HCAs. By acting as Maillard reaction intermediates, these compounds are highly likely to contribute to the chemical conversion of precursor amino acids and creatine into HCAs (Deng et al., 2024; Soladoye et al., 2017). However, previous literature indicates that the interaction between oxidative carbonyls and HCA pathways can be nonlinear and compound-specific. At moderate concentrations, reactive aldehydes facilitate the formation of key Maillard intermediates, thereby promoting the generation of specific HCAs. Conversely, at excessively high concentrations, these reactive carbonyls can compete with Maillard intermediates and consume available precursors, which can instead exert an inhibitory effect on certain HCA types depending on the chemical matrix (Zhao et al., 2025).

In summary, while lipid oxidation was largely influenced by storage temperature, protein oxidation showed a more prominent cumulative effect over the storage duration. These oxidative changes alter the internal reaction environment of the meat before cooking. Although extreme oxidative conditions can theoretically induce competitive inhibition as noted in the literature, our findings suggest that under practical storage conditions, these cumulative oxidative changes predominantly serve as indirect facilitators that prime the precursor matrix, thereby promoting HCA formation during subsequent cooking.

### 3.3 Changes in free amino acid composition

This study aimed to analyze changes in FAA composition under different storage conditions and to evaluate how these changes relate to HCA formation patterns. FAAs are well known as key precursors of the Maillard reaction and HCA formation; however, whether their changes during storage consistently correlate with HCA formation has not been clearly elucidated.

The changes in FAA composition are presented in Table 2. Depending on storage duration and temperature, distinct interaction effects were observed for each amino acid type. The contents of arginine, phenylalanine, and methionine were significantly highest on Day 14 of refrigerated storage (*P* < 0.05). In contrast, lysine showed its highest concentration on Day 1 of refrigerated storage (*P* < 0.05). Under frozen storage at Day 14, tyrosine and asparagine reached their highest levels (*P* < 0.05), while histidine content was significantly the lowest (*P* < 0.05).

**Table 2:** Changes in free amino acid composition (mg/100 g of sample) of chicken breast during refrigerated and frozen storage.

Storage	Days	Tyrosine	Histidine	Asparagine	Lysine	Arginine	Phenylalanine	Methionine	Proline
Refrigerated	1	3.01^B^	14.70^A^	9.20^B^	31.50^A^	8.67^B^	13.30^AB^	9.76^B^	32.73
	14	3.31^B^	16.35^A^	10.48^B^	16.95^B^	21.07^A^	15.10^A^	11.60^A^	28.88
Frozen	1	1.00^C^	14.63^A^	10.06^B^	18.69^B^	1.06^C^	12.95^B^	10.01^B^	29.74
	14	12.38^A^	7.43^B^	15.45^A^	20.45^B^	14.79^AB^	10.26^C^	6.82^C^	16.77
SEM		0.33	1.11	0.06	1.41	1.12	0.45	0.20	1.64

Values are expressed as mean values.

Different superscript letters within each amino acid indicate significant differences among storage condition × storage period (*P* < 0.05).

SEM, standard error of the mean. All values are expressed on a fresh meat weight basis.

Most FAAs showed an increase in content over time. This is consistent with previous reports that proteolysis, driven by protease activity during refrigerated storage, leads to the gradual accumulation of specific amino acids (Nishimura, 2010; Toldra, 1998). Conversely, the tendency of lysine to decrease after peaking on Day 1 of refrigeration (*P* < 0.05) is attributed to its high reactivity, leading to its consumption as storage progresses (Toldra & Flores, 1998). The significant increase in tyrosine and asparagine on Day 14 of frozen storage (*P* < 0.05) suggests that proteolysis under freezing conditions differs from that under refrigeration, resulting in a distinct FAA profile. These results align with previous studies indicating that structural changes in proteins during frozen storage can influence the FAA composition (Leygonie et al., 2012; Xia et al., 2009). On the other hand, the significant decrease in histidine under the same conditions (*P* < 0.05) may be explained by its structural vulnerability. Histidine contains an imidazole ring that makes it highly vulnerable to oxidative modification, resulting in a net structural loss (Estévez, 2011).

FAAs serve as direct nitrogen and carbon sources for HCAs through the Maillard reaction and Strecker degradation during heating (Skog et al., 1998). Specifically, phenylalanine and tyrosine, which contain aromatic ring structures, can contribute to the formation of PhIP and certain aminoimidazo-azaarene-type HCAs. Histidine, which contains an imidazole ring, is closely associated with the generation of IQ- and IQx-type HCAs. The increases in arginine and lysine observed in this study imply a potential to facilitate IQ-type HCA generation by reacting with creatinine during heating. Furthermore, the remarkable increase in tyrosine during frozen storage suggests that the potential to generate HCAs with aromatic structures may increase as storage duration increases.

In summary, the FAA composition changed significantly with storage temperature and duration, serving as a regulatory factor in the availability of key precursor amino acids involved in HCA formation. Therefore, storage conditions should be considered a critical factor that modulates the potential for HCA formation at the pre-cooking stage.

### 3.4 Changes in heterocyclic amine formation

This study evaluated the effects of storage temperature and duration on the formation patterns of HCAs in meat. Specifically, by comparing the generation trends of different HCA types according to storage conditions, we aimed to understand the impact of refrigeration and freezing on HCA formation pathways.

The changes in HCA content are presented in Table 3. Most HCAs exhibited significant changes in content under different storage conditions, and significant interaction effects were observed. Under refrigerated storage, as storage duration increased from 1 to 14 days, the concentrations of MeIQx, 7,8-DiMeIQx, PhIP, Harman, Norharman, IQ, and IQx increased significantly. Notably, the PhIP content approximately doubled from Day 1 to Day 14 under refrigeration. IQ and IQx also showed significant increases, indicating that extended refrigerated storage promotes the accumulation of HCAs. In contrast, under frozen storage, most HCAs showed no significant differences in their formation patterns. Only Norharman exhibited a significant increase with prolonged frozen storage (*P* < 0.05). Additionally, AαC and MeAαC were detected only under specific conditions, and no substantial changes were observed regarding storage temperature or duration for these compounds. Interestingly, despite lower oxidation markers under frozen storage, baseline PhIP levels remained relatively high at both Day 1 and Day 14. This observation aligns with established mechanisms, which report that PhIP formation in meat matrices can occur independently of storage-induced lipid or protein oxidation, relying instead on the direct thermal degradation of its abundant baseline precursors, phenylalanine and creatinine (Jägerstad et al., 1998). (See Table 4.)

**Table 3:** Changes in heterocyclic amine composition (ng/g dry matter) of chicken breast during refrigerated and frozen storage.

Storage	Days	MeIQx	4,8-DiMeIQx	7,8-DiMeIQx	PhIP	Harman	Norharman	Aac	MeAac	IQ	IQx
Refrigerated	1	1.80^C^	1.57	1.66^B^	15.05^B^	6.06^B^	6.20^BC^	ND	ND	6.90^B^	2.04^B^
	14	3.11 ^A^	2.15	2.92 ^A^	28.33 ^A^	14.46 ^A^	12.61 ^A^	1.07 ^A^	2.10 ^A^	23.52 ^A^	11.41 ^A^
Frozen	1	2.36^AB^	1.42	0.39^C^	29.58 ^A^	9.09^AB^	5.29^C^	ND	1.66^B^	10.19^B^	2.65^B^
	14	2.76^AB^	1.88	0.60^C^	36.70 ^A^	11.87^AB^	9.93^AB^	0.62^B^	1.14^B^	8.62^B^	3.27^B^
SEM		0.30	0.39	0.14	1.71	1.22	0.69	0.08	0.17	2.28	1.09

Values are expressed as mean values.

Different superscript letters within each heterocyclic amine indicate significant differences among storage condition × storage period (*P* < 0.05).

ND, not detected.

SEM, standard error of the mean.

**Table 4:** Changes in Volatile compound composition (μg/kg) of chicken breast during refrigerated and frozen storage.

Storage	Days	Hexanal	Heptanal	Octanal	Nonanal	Decanal	2-Heptenal (E)	2-Octenal (E)	2,4-Decadienal (E,E)	Tetradecanal	1-Hexanol	1-Heptanol	1-Octen-3-ol	2,3-Octanedione	2-Pentylfuran	Benzaldehyde
Refrigerated	1	240.54 ^A^	21.64^C^	35.85 ^A^	92.23^C^	4.85^B^	5.82 ^A^	15.40 ^A^	9.17 ^A^	5.17 ^A^	9.20 ^A^	17.78^C^	42.09^AB^	37.73	7.39^B^	7.54 ^A^
	14	68.41^D^	18.86^D^	28.88^B^	60.35^D^	2.86^C^	ND	ND	2.61^B^	1.82^B^	2.75^D^	13.82^D^	27.71^B^	ND	ND	4.79^B^
Frozen	1	211.26^B^	29.55^B^	24.59^C^	156.21 ^A^	7.62 ^A^	3.09^B^	7.63^B^	6.37^AB^	4.71^AB^	6.97^B^	28.24 ^A^	66.89 ^A^	32.64	10.77 ^A^	10.47 ^A^
	14	133.82^C^	34.64 ^A^	22.68^C^	144.80^B^	7.31 ^A^	2.19^C^	7.23^B^	3.12^B^	2.23^AB^	4.53^C^	24.70^B^	53.52^AB^	34.51	5.69^B^	4.08^B^
SEM		2.47	0.49	0.88	2.53	0.18	0.25	0.90	1.06	0.71	0.30	0.57	8.18	0.95	0.62	0.59

Values are expressed as mean values.

Different superscript letters within each heterocyclic amine indicate significant differences among storage condition × storage period (*P* < 0.05).

ND, not detected.

SEM, standard error of the mean.

In this context, this apparent discrepancy between the decreased total free phenylalanine in raw frozen matrices (Table 2) and the peak PhIP concentration (36.70 ng/g) observed on frozen Day 14 likely arises from the freeze-thaw sequence, which forces precursors to migrate outward and accumulate densely at the meat surface. The Maillard-mediated pathways driving HCA synthesis are primarily concentrated at the meat surface environment in direct contact with the heat source. Mechanical disruption of cell membranes by ice crystals formed during frozen storage creates discrete drip channels. During thawing and early thermal application, these channels potentially facilitate the outward migration of internal free phenylalanine, carrying it along with the moisture flux toward the thermal processing surface (Huff-Lonergan & Lonergan, 2007; Skog et al., 1998; Wu et al., 2021). Once at the surface, phenylalanine tends to stay behind rather than washing away with the drip, because its hydrophobic side chain binds readily to the exposed meat proteins (Damodaran & Parkin, 2017; Wu et al., 2021). Furthermore, PhIP can form directly through the thermal reaction between phenylalanine and creatinine, without needing reducing sugars like glucose (Murkovic, 2004; Skog et al., 1998). Consequently, although the overall amount of phenylalanine in the meat had decreased, its movement to the surface during freezing and thawing is suggested to maintain an abundance of this precursor directly at the thermal interface. This surface surplus, further concentrated as water evaporated during cooking, is believed to have sharply accelerated PhIP formation.

In contrast, under the same mass transfer environment, other polar HCAs (including MeIQx, 4,8-DiMeIQx, 7,8-DiMeIQx, IQ, and IQx) exhibited a declining or stagnant trend, likely due to precursor washout effects. The principal precursors of these species, such as glycine, alanine, threonine, and reducing glucose, are highly water-soluble and thus prone to dissolution and subsequent loss from the meat matrix via exudative drip (Wu et al., 2021). Therefore, the formation of these HCAs was likely suppressed because these highly water-soluble amino acids and glucose were washed away with the expelled drip. Consequently, while frozen storage induces a unique redistribution of precursors, the overall progressive accumulation of total HCAs in this study appears to remain closely linked with storage temperatures.

These findings generally align with previous reports suggesting that HCA content can increase with extended storage periods (Persson et al., 2008; Skog et al., 1998). Prior studies have noted that such changes accumulate more progressively under refrigerated conditions (Toldra, 1998; Zamora & Hidalgo, 2005). In contrast, the limited formation of certain IQ and IQx types under frozen storage in this study suggests that freezing may inhibit the mobility or reactivity of precursor substances (Persson et al., 2008). This is consistent with previous research suggesting that changes in moisture during freezing may restrict specific reaction pathways (Leygonie et al., 2012; Xia et al., 2009).

Furthermore, the limited detection of AαC and MeAαC likely reflects their sensitivity to specific precursor combinations or reaction conditions (Felton & Knize, 1991; Murkovic, 2004). The HCA formation patterns under different storage conditions appear to be closely related to the degree of oxidative reactions during storage. Specifically, the significant increase in multiple HCAs (MeIQx, 7,8-DiMeIQx, PhIP, Harman, Norharman, IQ, and IQx) during extended refrigerated storage reflects the consequences of ongoing lipid and protein oxidation. This process progressively establishes a precursor environment, which is defined by the reactive matrix of accumulated carbonyls and liberated free amino acids. Our findings demonstrate that carbonyl and dicarbonyl compounds progressively accumulate during refrigerated storage through lipid and protein oxidation, coinciding with altered free amino acid profiles that correlate with the statistical increase in subsequent HCA formation. While earlier literature confirms that model carbonyl compounds can facilitate HCA synthesis via the Maillard reaction and Strecker degradation (Skog et al., 1998; Zamora & Hidalgo, 2005), the present study clarifies that this storage-induced accumulation is closely associated with the increased HCA generation in actual muscle food matrices.

In summary, this study's results suggest that oxidative reactions during refrigerated storage are a major factor in increasing the potential for HCA formation. Conversely, frozen storage effectively limits the accumulation of most individual HCAs over time by suppressing these oxidative processes, except for specific species like PhIP.

### 3.5 Multivariate analysis (PCA and biplot)

**Fig. 3:**
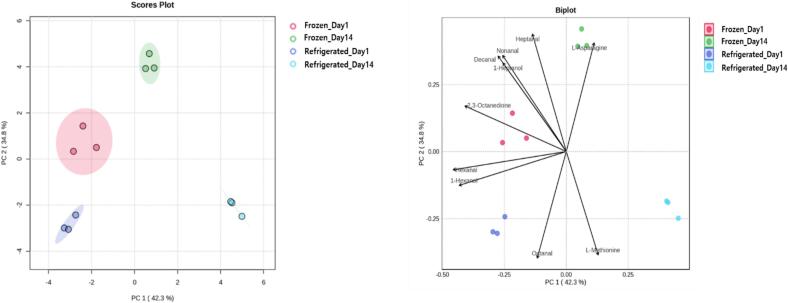
Principal component analysis of chicken breast based on free amino acids and volatile compounds during refrigerated and frozen storage.

The present study conducted a comprehensive evaluation of HCA contents, volatile compounds, and free amino acid composition as a function of storage temperature and duration. Specifically, under the hypothesis that the relative contributions of HCAs, volatile compounds, and amino acids vary with storage conditions, PCA was used to examine differences between treatment groups at multivariate level. The PCA results are presented in Fig. 3. The first two principal components, PC1 and PC2 explained 42.3% and 34.8% of the total variance, respectively, with a cumulative explanatory power of 77.1%. In the PCA score plot, the samples were clearly separated based on storage temperature and duration. Samples from Day 1 and Day 14 were distinctly partitioned, and their positions shifted depending on refrigerated or frozen conditions, even within the same storage duration. This indicates that variations in storage conditions significantly influenced the overall chemical composition.

Analysis of the variables in the biplot revealed that compounds such as hexanal, nonanal, 2,3-octanedione, and octanal were clustered in a similar direction; these are representative products of lipid oxidation. Day 1 samples, characterized by shorter storage duration, were distributed farther from these oxidation-related compounds. In contrast, Day 14 samples, in which oxidation had progressed further, tended to shift toward these oxidative markers. This suggests that as storage progresses, the volatile composition within the samples gradually changes, with the influence of oxidation-related components becoming more pronounced.

Distinct differences were also observed according to the storage method. At the same storage duration, refrigerated samples were positioned farther along the axis of change than frozen samples. This aligns with general storage characteristics, in which lower temperatures result in slower chemical changes. Notably, both frozen and refrigerated storage samples moved along a similar trajectory, suggesting that the storage method is strongly associated with the magnitude of these chemical shifts rather than altering their overall directionality.

In summary, the PCA and biplot results effectively visualize distinct covariance patterns, illustrating how the multivariate profiles of the samples progressively shift in relation to storage duration and method. As storage duration increases and temperature rises, the composition of the samples shifts progressively toward oxidation-related compounds, resulting in the distinct separation patterns observed in this study.

### 3.6 Variable importance in projection analysis

Following the PCA and biplot analyses, which confirmed clear discrimination of meat chemical characteristics by storage temperature and duration, a VIP analysis was conducted. This analysis aimed to identify the key variables driving the separation between treatment groups within the multivariate model, including HCA contents, volatile compounds, and free amino acid profiles. The objective was to support the separation observed in the PCA quantitatively and to identify the primary compounds that explain differences in HCA formation across storage conditions. Prior to identifying these key features, the reliability of the 3-component PLS-DA model was rigorously evaluated. The model exhibited an excellent goodness-of-fit and predictability, with a cumulative *R*^2^ of 0.99 and a *Q*^2^ of 0.96. Furthermore, a 100-time permutation test yielded an empirical *P*-value of *P* < 0.01 (0/100), confirming the strong statistical robustness and significance of the model without overfitting.

**Fig. 4:**
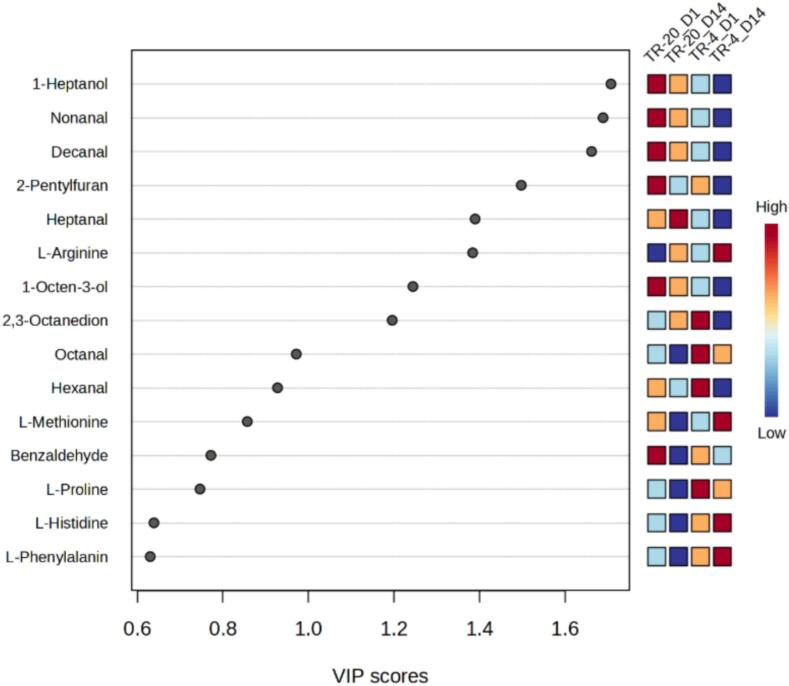
VIP scores of free amino acids and volatile compounds associated with heterocyclic amine formation during refrigerated and frozen storage of chicken breast meat.

The results of the VIP analysis are presented in Fig. 4, confirming that numerous variables with VIP scores greater than 1.0 significantly contributed to the separation between the treatment groups. In particular, lipid oxidation-derived volatile compounds, such as 1-heptanol, nonanal, decanal, 2-pentylfuran, and heptanal, exhibited high VIP values, acting as major variables in explaining the oxidative differences across storage conditions. Additionally, volatile carbonyls including hexanal, octanal, and 2,3-octanedione showed relatively high VIP scores, indicating that changes in the composition of carbonyl compounds driven by storage conditions played a leading role in discriminating between the treatment groups.

In summary, the VIP analysis demonstrates that the separation between treatment groups based on storage conditions is not merely a simple chemical difference but is explained by variations in the reaction environment established before HCA formation. The variations in lipid oxidation-derived carbonyls and free amino acid compositions that developed during storage served as potential precursors associated with the pre-cooking reaction environment, exhibiting a strong correlation with subsequent HCA generation patterns. These findings imply that HCA formation is not an exclusive phenomenon of heating but instead is closely linked to the comprehensive chemical environment established during storage. This suggests that storage conditions serve as a critical regulatory factor in determining the risk of HCA formation.

## 4 Conclusion

This study demonstrates the biochemical shifts in chicken breast across varying storage temperatures and durations and shows that these shifts play a pivotal role in subsequent HCA formation. Our findings demonstrate that refrigerated storage leads to a significant accumulation of lipid and protein oxidation products, which are characterized by substantial alterations in volatile carbonyl profiles, while simultaneously modulating the FAA profile, particularly L-arginine and *L*-phenylalanine. These storage-induced transitions modify the precursor matrix into a highly reactive environment of accumulated carbonyls and free amino acids that favor HCA synthesis during subsequent thermal processing. Conversely, frozen storage effectively arrests these oxidative and proteolytic trajectories, thereby mitigating overall HCA generation potential except for specific species like PhIP. Our results imply that HCA formation is not driven solely by cooking conditions but is strongly associated with the chemical environment pre-established during storage. Consequently, storage conditions should be recognized as a strategic window for intervention, providing a scientific basis for developing optimized processing methods to mitigate the carcinogenic risk of cooked chicken meat products. Building upon this, the present study introduces a proactive approach to food safety by establishing storage-phase chemical properties as predictive indicators for monitoring and mitigating heterocyclic amine formation in cooked chicken products.

## CRediT authorship contribution statement

**Jeong-Uk Eom:** Writing – original draft, Visualization, Formal analysis, Data curation. **Nayeem Mia:** Data curation, Resources. **Jin-Kyu Seo:** Visualization, Validation, Methodology, Data curation. **Han-Sul Yang:** Writing – review & editing, Supervision, Conceptualization.

## Declaration of competing interest

The authors declare that they have no known competing financial interests or personal relationships that could have appeared to influence the work reported in this paper.

## Data Availability

Data will be made available on request.
